# The Influence of Tai Chi Exercise on Proprioception in Patients with Knee Osteoarthritis: Results from a Pilot Randomized Controlled Trial

**DOI:** 10.5772/57137

**Published:** 2013-10-04

**Authors:** Anna Schmid, Timothy McAlindon, Christopher H. Schmid, Chenchen Wang

**Affiliations:** 1 Center for Complementary and Alternative Medicine, Division of Rheumatology, Tufts Medical Centre, Tufts University School of Medicine, Boston, MA, USA; 2 Centre for Evidence-Based Medicine, Brown University, Providence, RI, USA

**Keywords:** Tai Chi, Exercise, Proprioception, Knee Osteoarthritis

## Abstract

**Purpose:**

Previous long-term observational studies found that Tai Chi practitioners had better knee-joint proprioceptive acuity versus controls in an older population. We evaluated the effects of Tai Chi for knee-joint proprioception in knee osteoarthritis (OA) in a randomized controlled trial.

**Methods:**

We randomized 40 eligible individuals (age > 55, BMI ≤ 40 kg/m^2^ with knee pain on most days of the previous month and tibiofemoral OA (K/L grade ≥2) to Tai Chi (10 modified forms from classical Yang style) or to an attention control (stretching and wellness education). The 60 minute intervention sessions occurred twice-weekly for 12 weeks. The knee joint proprioception was measured using a Biometrics™ electrogoniometer with an ADU301 angle display unit during each assessment visit. Three test angles (30, 45 and 60 degrees) were evaluated with each subject in a sitting position taken as neutral (0 degrees). The mean error (absolute angle error) between the actual and replicated angles was calculated for each of the three test angles. The Tai Chi and control groups were compared by intention-to-treat using t-tests.

**Results:**

The participants had a mean age of 65 y (SD 7.8), a mean disease duration of 10 y (SD 7.6), a mean BMI of 30.0 kg/m^2^ (SD 4.8), and median K/L grade 4; 75% were female, 70% were white. The participants in the Tai Chi intervention exhibited significantly improved proprioception at 30 degrees, but not at 45 or 60 degrees, at 12 weeks. Patients who continued Tai Chi practice after 12 weeks also reported no significant improvements in knee proprioception at 24 and 48 weeks.

**Conclusion:**

Tai Chi appears to be beneficial for knee proprioception in people with severe knee OA at a 30 degree test angle immediately following 12 weeks of practice. However, we were unable to demonstrate that Tai Chi has any long-term effects on knee proprioception, nor were we able to find any effects on proprioception at larger test angles (45 and 60 degrees). Standardized and reproducible measures for knee proprioception should be explored in future research.

## 1. Introduction

Knee osteoarthritis (OA) is an increasing problem in the elderly population, resulting in pain, functional limitations, disability, reduced quality of life, and substantial direct and hidden healthcare costs.^1–3^ The pathophysiological basis of knee OA is multifaceted and includes the degeneration of articular structures and impaired muscle function, and psychological traits like chronic pain.^4–6^ To date, feasible preventive intervention strategies or effective disease-modifying remedies for knee OA do not exist.

Neurological deficits, such as quadriceps sensory dysfunction (i.e., proprioceptive acuity) are proposed as a factor in the progression of knee OA. Proprioception - or the perception of limb position in space - is critical to the preservation of joint stability. Impaired proprioception may play a role in the progression of knee OA by causing abnormal stresses on the knee joint. These stresses can produce changes in the articular structures, which may result in decreased confidence in postural stability and, consequently, impaired performance in daily activities. Proprioception is well-documented to decrease with the natural ageing process, but decreases are more profound in patients with knee OA.^7–9^ In a recent study conducted by Baert et al.,^10^ proprioceptive deficits were observed in women with established - but not early - knee OA, implying that proprioception may be a consequence of structural degeneration rather than a risk factor in the pathogenesis of knee OA.

Research suggests that exercise may be an effective therapy for promoting proprioceptive acuity.^11–13^ Hurley and Scott found that exercise significantly improved quadriceps muscle strength and knee joint position sense in 52 patients with knee OA completing an exercise regime. Moreover, these improvements were maintained in the exercise group after six months of follow-up.^11^ Tai Chi, with an emphasis on balance, muscle strengthening and the integration of the mind and body, may be an ideal proprioceptive exercise for older individuals with knee OA.^12,13^ Tai Chi has been shown to act on a number of sensorimotor systems that contribute to postural control. A longitudinal study conducted by Tsang and Hui-Chan reported that 21 elderly individuals practicing Tai Chi for at least three years had better knee-joint proprioceptive acuity (less absolute angle errors in passive knee joint repositioning testing) than the 21 controls.^12^ The research group went on to report that in a group of 68 elderly subjects, those practicing Tai Chi regularly for a minimum of four years showed significantly better knee joint proprioception than those subjects who were long-term swimmers or runners or sedentary controls.^12^ Despite limited observational evidence, these results generally support the view that long-term Tai Chi practice led to better knee-joint proprioceptive acuity and neuromuscular activities in an older population.

Thus, we aimed to evaluate the effectiveness of Tai Chi on knee proprioception in a group of older individuals with knee OA. We hypothesized that participants receiving Tai Chi would show greater improvement in knee joint proprioception than participants treated with an attention control intervention consisting of wellness education and stretching.

## 2. Methods

### 2.1 Setting and Participants

The study was conducted at Tufts Medical Centre, an urban tertiary care academic hospital in Boston, USA. The detailed methodology of the full study protocol was published in a previous publication.^14^ Patients with knee OA were recruited from the greater Boston area and written consent was obtained from all eligible participants prior to enrolment.

To ensure the adequate enrolment of underrepresented groups, we placed advertisements in local media. We also used the rheumatology clinic patient database at Tufts Medical Centre to identify patients with knee OA. For interested respondents, we determined eligibility through a brief, scripted interview which posed questions whose predictive values for knee OA were known from population-based data. Applicants who screened positive on the telephone interview were scheduled for eligibility visits, whereby written informed consent was obtained.

The eligibility criteria were: age ≥ 55 years; body mass index (BMI) ≤ 40 kg/m^2^; WOMAC pain subscale score >40^15^ (with a range 0–100); fulfilment of the American College of Rheumatology criteria for knee OA^16^ with radiographic knee OA grade ≥ 2.^17^ We excluded individuals with prior Tai Chi training or experience with similar types of alternative medicine, such as Qi Gong or yoga; individuals with serious medical conditions limiting their ability for full participation as determined by their primary care physicians; individuals with intra-articular steroid injections given in the previous three months; individuals with reconstructive surgery on the affected knee or any intra-articular hyaluronate injections in the previous six months; and individuals unable to pass the Mini-Mental examination with a score < 24.^18^ For individuals with knee OA in both knees, the knee reported as the most painful at the baseline was designated as the study knee. If both knees were equally affected (occurring in two participants) one knee was chosen at random as the affected knee.

### 2.2 Randomization

The participants were randomly assigned to Tai Chi (n=20) or an Attention Control group (n=20). Randomization assignments were designated by the statistician (CS) using computer-generated numbers to randomize permuted blocks of size two and four so that each block was complete. They were provided in sealed, opaque envelopes and opened upon the participant’s agreement to participate. The block size was randomly assigned to minimize the correct prediction of assignments while preserving approximate balance between groups. Outcomes that required the analysis of knee strength were based on the evaluation of the knee reported as most painful at the baseline. If both knees were equally affected (which occurred in two participants), one knee was chosen at random as the affected knee.

### 2.3 Interventions

Subjects in the Tai Chi group participated in 60 minute Tai Chi sessions twice weekly for 12 weeks. The sessions were instructed by a Tai Chi master with more than 20 years of teaching experience and included: (1) 10 minutes of self-massage and a review of Tai Chi principles; (2) 30 minutes of Tai Chi movements; (3) 10 minutes of breathing technique; (4) 10 minutes of relaxation. The programme consisted of ten forms from classical Yang Style Tai Chi^19^ with minor modifications that were suitable for people with knee pain. The modifications involved eliminating stances that required greater than 90 degrees of knee flexion and which could cause excess knee joint stress. The patients were instructed to practice Tai Chi daily at home and to maintain their usual physical activities throughout the study.

Subjects in the attention control group received a wellness education and stretching programme.^14,20^ The control group attended 60 minute sessions twice weekly for 12 weeks. Every session included 40 minutes of didactic lessons on: (1) knee OA as a disease; (2) diet and nutrition; (3) therapies to treat knee OA; (4) physical and mental health education (e.g., recognizing and dealing with stress). The final 20 minutes consisted of stretching exercises involving the upper body, trunk and lower body, with each stretch being held for 10 to 15 seconds. The participants were instructed to practice at least 20 minutes of stretching exercises per day at home and to maintain their usual physical activities.

### 2.4 Measurement of Proprioception

Knee joint proprioception was measured at each assessment visit (baseline, 12, 24, 48 weeks) with a non-weight-bearing passive knee joint repositioning test using a biometrics electrogoniometer with an ADU301 angle display unit. Three test angles (30, 45, and 60 degrees) were evaluated with each participant in a sitting position taken as neutral (0 degrees). The participant was instructed to sit on the edge of an examination table with their lower leg dangling freely over the edge of the table. The electrogoniometer was then placed longitudinally in alignment with the participant’s femur and tibia and was secured to the skin with double-sided medical tape. With the participant’s eyes closed, the examiner first moved the participant’s leg into one of the test angles and held it for five seconds before returning the leg to the freely dangling position. With their eyes still closed, the participant was asked to attempt to reproduce the test angle. The practiced and replicated angle was repeated for all three test angles. The mean error (absolute angle error) between the actual and replicated angles was calculated for each of the three test angles.

The electrogoniometer was placed longitudinally in alignment with the femur and tibia with double-sided medical tape and used to determine each of the three test angles. The participants were first shown one of the angles, which was held for a few seconds, and were then asked to close their eyes and attempt to reproduce the angle; this was repeated for all three test angles. We also repeated these assessments at 24 and 48 weeks to test the durability of the response.

## 3. Statistical Analysis

We analysed the data on an intent-to-treat basis and compared between the groups any changes in proprioception across all times at the baseline, 12, 24, and 48 weeks with mixed models, using ‘time’ and ‘group’ as categorical fixed factors with random intercepts and first-order autocorrelation of the errors. The mean error (absolute angle error) between the actual and replicated angles was calculated for each of the three test angles. The Tai Chi and control groups were compared by intention-to-treat using t-tests. We evaluated potential effects of confounding factors or interaction with treatment by covariates including age, gender, BMI, disease duration, disease severity (pain, function and radiographic score), co-morbidities, health status, effusion and function, modified Tai Chi style, changes in joint position and body weight, and use of pain medications. A two-sided p-value < 0.05 was considered to indicate statistical significance. The results were presented as between group differences.

## 4. Results

**Figure 1:**
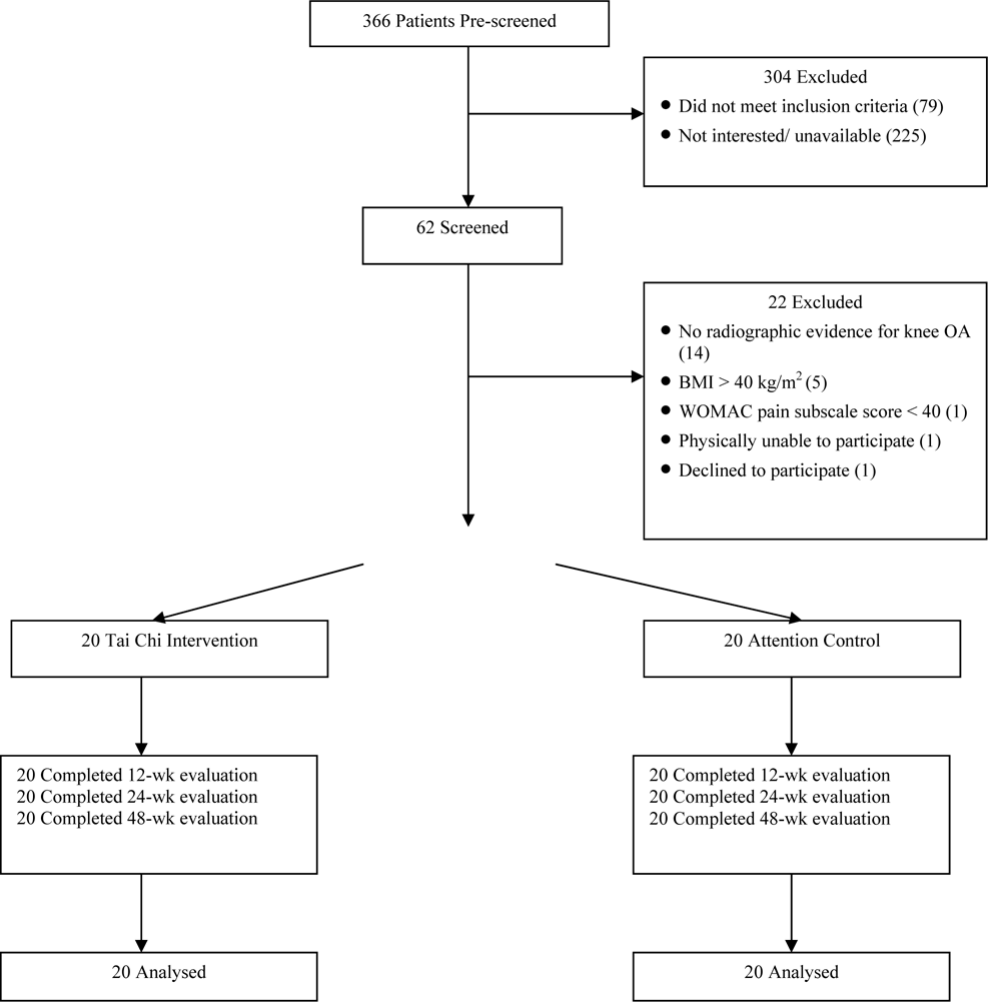
Study Flow Chart

Between October 2005 and February 2006, 366 individuals were screened by telephone and 62 were identified for further evaluation. Forty participants (65%) were found eligible and randomized to the Tai Chi or Attention Control group (See Figure 1). The remaining participants were excluded for a variety of reasons, the major one being the absence of radiographic evidence of knee OA. The main results of the trial were reported in a previous publication.^21^

Table 1 shows the baseline data of the 40 participants before randomization to the intervention groups. The 40 participants had a mean age of 65 years (±7.8 years), a mean disease duration of 10 years (± 7.6 years), a mean BMI of 30.0 kg/m^2^ (± 4.8 kg/m^2^), and a median K/L grade of 4; 75% were female, 70% were white. No significant differences were seen at the baseline demographics, radiographic score or outcome measures. The attendance rate was 85% for Tai Chi versus 89% for the attention control.

Table 2 shows the results of changes in proprioception scores. After 12 weeks, the participants in the Tai Chi group exhibited significantly improved proprioception compared to the control group at 30 degrees [−2.53 (5.22) versus 2.11 (5.64), p=0.01], but no significant differences were demonstrated between the groups at the 45 and 60 degree test angles. Furthermore, there were no significant differences in proprioception between the groups at any of the three test angles at 24 and 48 week follow-up visits. This includes participants who continued Tai Chi practice after 12 weeks.

At the 24 week follow-up, 13 out of 20 participants (65%) in the Tai Chi group continued to practice Tai Chi while 12 out of 20 (60%) in the control group continued to practice stretching exercises. At the 48 week follow-up, these rates were 9 out of 20 (45%) for Tai Chi and 8 out of 20 (40%) for the control.

## 5. Discussion

Our findings suggest that Tai Chi may be beneficial for knee proprioception in patients with severe knee OA at a 30 degree test angle immediately following 12 weeks of practice. However, we were unable to demonstrate that these effects are maintained at follow-up. There have been several previous trials testing the effects of Tai Chi for joint proprioception in patients with knee OA.^10–14^ Our results are partially consistent with the results of two observational studies examining the impact of Tai Chi on knee-joint proprioception and neuromuscular activities.^12,13^ However, it is important to note that our results were likely limited by problems we encountered in measuring proprioception. These problems included limitations with the instrument used to measure proprioception itself, with the methods we employed in using the instrument to measure proprioception, and with the length of the Tai Chi intervention that study participants received. In this section, we aim to explain the methodological challenges in measuring knee proprioception in order to improve these measurements for future research. Table 3 summarizes the methodological challenges that we confronted.

The first methodological challenge may be attributed to the use of an electrogoniometer - rather than a custom-built device - to measure proprioception. Measurement by the electrogoniometer can be affected by the skin and body compositions of the study population. Furthermore, the device is dependent upon proper placement by the examiner, highlighting the importance of extensive training for the testers placing the markers to limit variability.

A second methodological challenge may be attributed to the protocol design, in particular the knee selection, reference joint angle and testing angle. The study knee, designated by the presence of knee OA or a higher pain index, was tested when in fact the ‘kicking knee’ is most commonly tested for proprioception acuity. Other neuromuscular research suggests testing proprioception in the ‘stabilizing leg’ because it is the postural support limb. Our testing procedures defined the natural resting position of the participants to be zero degrees and neglected to account for variability in the resting positions of the participants, particularly among Knee OA patients who experience varying degrees of structural changes.^22^ More importantly, the resting position may change over time with each participant, particularly while undergoing an intervention such as Tai Chi, which focuses on joint alignment. The test angles measured were 30, 45 and 60 degrees. Patients, especially those with severe knee OA, may have a full knee extension of less than 60 degrees and, thus, would be limited in their testing. Therefore, test angles, along with the resting position angle, ought to be calculated on an individual basis from an anatomical position designated as ‘zero degrees’. Furthermore, only one trial was tested for the each of the three test angles. Increasing the number of trials tested would ensure more accurate calculations.

Thirdly, our proprioception outcomes may have been influenced by the procedures implemented. To begin testing, the examiner moved the participant’s leg to the test angle, held the angle for three seconds, and then lowered the leg to rest. Next, the participant was instructed to close their eyes and reproduce the test angle. The discrepancy between passive positioning and active repositioning, as well as between the opened and closed eyes, raises concerns for the accuracy of measurement. Strong proprioception research relies on passive positioning and repositioning with closed eyes throughout the entirety of the measurement procedures in order to minimize confounding variables and participant distractions. Our results may have been strengthened if the participants were given the opportunity for practice trials, if they were tested both with their eyes opened and closed, and if more trials were performed.

Finally, we cannot rule out the possibility that the Tai Chi performed in this clinical trial may have constituted an insufficient amount of exercise in influencing proprioceptive acuity. Proprioceptive gains are specific to the joint angles trained, but participants in our programme practiced a modified Yang style Tai Chi that eliminated deep knee bends in order to project the knees. By limiting the joint movements at the knee during the training, it is possible that we also limited the benefits to proprioceptive acuity at the knee. Additionally, the participants practised Tai Chi for just 12 weeks and had no prior experience with Tai Chi or similar disciplines prior to the intervention. In contrast, previously mentioned observational studies found significant effects of Tai Chi on proprioception in participants who were termed ‘Tai Chi practitioners’ with a minimum of three years’ experience.^12,13^ In addition, the small sample size may have limited the data’s interpretation and the evaluation of the results.

In conclusion, while we were able to demonstrate that Tai Chi may benefit knee proprioception in patients with severe Knee OA under certain conditions, we were unable to replicate the results of several previous clinical trials finding that proprioceptive deficits in older patients with knee OA consistently improved following an exercise intervention. As impaired proprioception may play a role in the progression in knee OA, finding feasible measures to improve proprioceptive acuity is of critical interest. We conclude that further research should focus on identifying standardized and reproducible methods for the accurate measurement of knee proprioception.

## Figures and Tables

**Table 1:** Baseline Characteristics of Study Participants*

Variable	Tai Chi (n=20)	Attention Control (n=20)	Total (n=40)
Demographics			
Female, no. (%)	16 (80)	14 (70)	30 (75)
Age, yr.	63 ± 8.1	68 ±7.0	65 ±7.8
White, no. (%)	14 (70)	14 (70)	28 (70)
> High school education, n (%)	20 (100)	19 (95)	39 (98)
Body Mass Index, kg/m^2^	30.0 ± 5.2	29.8 ± 4.3	29.9 ± 4.8
Disease condition			
Duration of knee pain, yrs. (on study knee)	9.7 (7.0)	9.7 (8.3)	9.7 (7.6)
Radiograph score, no. (%)			
K/L 2	4 (20)	3 (15)	7 (18)
K/L 3	7 (35)	3 (15)	10 (25)
K/L 4	9 (45)	14 (70)	23 (58)
Knee surgery, no. (%)	6 (30)	8 (40)	14 (35)
Knee Replacement, no. %	1 (5)	1 (5)	2 (5)
Patient VAS, 0–10 cm†	4.2 ± 2.1	4.8 ± 2.0	4.5 ± 2.0
Physician VAS (study knee), 0–10 cm †	4.8 ± 1.7	5.8 ± 2.2	5.3 ± 2.0
WOMAC-Pain, 0–500 mm†	209.3 ± 58.5	220.4 1 ± 101.0	214.8 ± 81.7
WOMAC-Physical Function, 0–1700 mm †	707.6 ± 246.9	827 ± 258.8	767.3 ± 256.9
WOMAC-Stiffness, 0–200 mm †	105.7 ± 37.3	120.7 ± 50.4	113.2 ± 44.4
Receiving NSAID prior to study, no. (%)	9 (45)	13 (65)	22.0 (55.0)
Receiving analgesics prior to study, no. (%)	4 (20)	6 (30)	10.0 (25.0)
Self-reported co-morbidities, no. (%)			
Congestive Heart Disease	1 (5)	4 (20)	5 (13)
Hypertension	7 (35)	12 (60)	19 (48)
Diabetes	0 (0)	4 (20)	4 (10)
Health-related quality of life and others			
SF-36 PCS, 0–100 ‡	37.5 ± 8.5	32.0 ± 8.8 §	34.8 ± 9.0
SF-36 MCS, 0–100 ‡	51.4 ± 12.2	50.8 ± 12.6	51.1 ± 12.3
CES-D, 0–60 †	13.6 ± 11.7	9.3 ± 9.2	11.5 ± 10.6
Self-Efficacy score, 1–5 ‡	3.1 ± 1.1	3.3 ± 1.0	3.2 ± 1.0
Outcome Expectation score, 1–5 ¶	4.1 ± 0.6	4.3 ± 0.4	4.2 ± 0.5
Physical Performance			
6-Minute Walk Test (yards)	500.1 ± 114.3 [19]	488.9 ± 109.2	494.3 ± 110.4 [39]
Balance score, 0–5	4.0 ± 0.7	3.8 ± 0.8	3.9 ± 0.7
Chair stand score (seconds)	40.8 ± 13.4	35.6 ± 9.2 [19]	38.3 ± 11.7 [39]

* Values are mean (SD) unless otherwise noted. N = 20 except where specified by data in square brackets. P values were calculated by the t-test for continuous variables and the chi-square test or Fisher exact tests for categorical variables. K/L = Kellgren and Lawrence scale; VAS = Visual Analogue Scale; WOMAC = Western Ontario and McMaster Universities; NSAID = Non-steroidal Anti-inflammatory Drugs; SF-36 = Short Form-36 questionnaire; CES-D = Centre for Epidemiology Studies Depression index.

† Lower scores indicate improved state.

‡ Higher scores indicate improved state.

§ P < 0.05

¶ Higher scores indicate high outcome expectations.

**Table 2:** Changes in Proprioception Scores

Variable	Mean Scores (SD)		Change from Baseline Mean (SD)		Total Number of Subjects	Tai Chi vs. Control P-value
	Tai Chi (N=20)	Control (N=20)	Tai Chi	Control		
**30 Degrees**						
Baseline	5.58 (4.15)	4.26 (2.88)				
Week 12	3.00 (2.55)	6.55 (4.38)	−2.53 (5.22)	2.11 (5.64)	40	**0.01**
Week 24	3.60 (4.58)	3.80 (2.50)	−2.58 (5.84)	−0.68 (2.60)	40	0.20
Week 48	4.10 (2.79)	4.79 (4.70)	−1.26 (4.75)	−0.11 (4.42)	39	0.40
**45 Degrees**						
Baseline	4.60 (4.03)	3.63 (3.08)				
Week 12	4.00 (3.29)	4.75 (4.12)	−0.60 (5.06)	0.95 (5.04)	40	0.30
Week 24	2.75 (2.59)	1.65 (1.73)	−1.85 (4.40)	−1.95 (3.27)	40	0.90
Week 48	2.75 (1.55)	3.00 (2.98)	−1.85 (4.52)	−1.22 (4.41)	39	0.70
**60 Degrees**						
Baseline	3.05 (3.05)	3.24 (2.93)				
Week 12	2.45 (2.44)	2.84 (2.61)	−0.60 (4.03)	0.06 (3.80)	39	0.60
Week 24	1.94 (2.46)	3.60 (3.31)	−1.18 (3.28)	−0.88 (3.76)	27	0.80
Week 48	3.21 (2.44)	1.94 (1.89)	0.26 (4.59)	−1.44 (4.11)	37	0.30

Participants in the Tai Chi arm exhibited significantly improved proprioception at 30 degrees, but not at 45 and 60 degrees, at 12 weeks (Table 2).

**Table 3:** Limitations in Measuring Proprioception Using Our Methodology

PROCEDURES	LIMITATIONS	SOLUTIONS
1. Electrogoniometer used to determine the test angles	Not sensitive enough to measure knee proprioception. Affected by the skin/body composition of the study population	Custom-build devices to measure proprioception
2. Electrogoniometer placed longitudinally in alignment with the femur and tibia, secured with tape	Variability in placement of electrogoniometer markers	Importance of training the examiner
3. Test knee determined by participants indicting his/her ‘kicking leg’	The ‘stabilizing leg’ may actually better represent postural support	Test both legs
4. Participant instructed to a neutral sitting position (0°): edge of table, lower leg dangling	Variability in resting position between participants and within participants over time	Calculate the resting positions on an individual basis and use the anatomical position as 0°
5. Examiner moved the participant’s leg to the 30° test angle, held for a few seconds, lowered back to rest. Participant then closed eyes and attempted to reproduce the 30° angle.	Discrepancy between passive positioning and active repositioning	Ensure that the methods of positioning and repositioning are consistent
6. After the first trial, participant asked to rest and then repeat active repositioning for a second trial at 30°	Two trials may not be enough	Increase the number of trials
7. Participants practiced modified form of Yang style Tai Chi	Limited joint movements may have limited effect on proprioceptive acuity	Increase intensity of knee bends or duration of practice
8. Participants only practiced Tai Chi for 12 weeks in the intervention	12 weeks may not be long enough to affect change in proprioceptive acuity	Measure gains in proprioception after longer Tai Chi intervention
